# Case Report: The effect of automated manual lymphatic drainage therapy on lymphatic contractility in 4 distinct cases

**DOI:** 10.3389/fmedt.2024.1397561

**Published:** 2024-07-17

**Authors:** Melissa B. Aldrich, John C. Rasmussen, Ron J. Karni, Caroline E. Fife, Frank Aviles, Kristen A. Eckert, M. Mark Melin

**Affiliations:** ^1^ Brown Foundation Institute of Molecular Medicine, McGovern Medical School, The University of Texas Health Science Center at Houston, Houston, TX, United States; ^2^ Division of Head and Neck Surgical Oncology, Department of Otorhinolaryngology, McGovern Medical School, The University of Texas Health Science Center at Houston, Houston, TX, United States; ^3^ Intellicure, LLC, The Woodlands, TX, United States; ^4^ Division of Geriatrics, Department of Internal Medicine, Baylor College of Medicine, Houston, TX, United States; ^5^ Lymphatic and Wound Healing Services, Hyperbaric Physicians of Georgia, Cumming, GA, United States; ^6^ Strategic Solutions, Inc., Bozeman, MT, United States; ^7^ Gonda Vascular Center, Wound Clinic, Mayo Clinic, Rochester, MN, United States

**Keywords:** manual lymphatic drainage, lymphedema, automated MLD therapy, complex regional pain syndrome, rheumatoid arthritis, pain

## Abstract

**Introduction:**

Automated manual lymphatic drainage therapy (AMLDT) is available for home use in the form of a pneumatic mat of 16 pressurized air channels that inflate and deflate to mimic the stretch and release action of manual lymphatic drainage therapy. Four cases (a patient with complex regional pain syndrome and lymphedema, a healthy patient, a breast cancer survivor with chronic pain, and a patient with a history of abdominal surgery) underwent near-infrared fluorescence lymphatic imaging (NIRFLI) with AMLDT to evaluate the effect of AMLDT on lymphatic pumping and pain.

**Methods:**

Each patient received 32–36 injections of 25 μg indocyanine green (ICG) on the anterior and posterior sides of their body and underwent 1 h of NIRFLI to assess the drainage of ICG laden lymph toward regional nodal basins at baseline. Each patient lay supine on the mat for 1 h of AMLDT with NIRFLI to assess lymphatic flow during treatment. A final NIFRFLI assessment was done 30–60 min posttreatment with the patient in the supine and prone position. Patients reported baseline and posttreatment pain using the Visual Analogue Scale. An imager analyzed NIRFLI images using ImageJ (US National Institutes of Health). Using time stamps of the first and last images to determine time lapsed and the number of pulses observed in a timeframe, pulsing frequency (pulses/min) was obtained to assess lymphatic function.

**Results:**

All 4 cases completed the NIRFLI and AMLDT without complications; all 3 patients with baseline pain reported reduced pain posttreatment. AMLDT appeared to alter lymphatic contractility, with both increased and decreased pulsing frequencies observed, including in nonaffected limbs. Pulsing frequencies were very heterogeneous among patients and varied within anatomic regions of the same patient.

**Discussion:**

This proof-of-concept study suggests that AMLDT may impact lymphatic contractility. Further research on its effect on lymphatic function is warranted.

## 1 Introduction

The lymphatic system is an essential part of the vascular system, with multitasking functions to absorb fats and fat-soluble nutrients in the digestive system; eliminate excess extravasated fluid from blood vessels as part of homeostasis; and facilitate immune responses by surveilling pathogens and enabling immune cell transit within lymph fluid (1–6). Dysfunctional lymphatic vessels and/or lymph nodes can result in build-up of lymph fluid in the interstitial space, resulting in lymphedema (LE), which initially manifests as edema in the extremities and progresses to fibrosis, with eventual impaired limb function, pain, depression, and increased cellulitis risk. From 2012 through 2017, there were more than 165,000 hospital admissions due to LE, 92% of which were associated with cellulitis in the United States (US) (7). While primary LE is genetic, most cases are secondary and related to obesity, or acquired following radiotherapy, surgery, trauma, or infections. Approximately 15.5% of breast cancer survivors develop LE, with 24% having LE 7 years postdiagnosis (2, 8, 9). Decreased lymph flow leads to the accumulation of proinflammatory cytokines that continuously activate nociceptors responsible for pain, resulting in a chronic inflammatory response that can be associated with chronic pain and disease (4–6). Chronic rheumatoid arthritis (RA) and complex regional pain syndrome (CRPS) appear to be associated with impaired lymphatic function (4, 10–12).

Therapy targeting LE aims to reduce lymphatic fluid in the affected limb to improve function and prevent progression (2, 13–15). The most widely accepted treatment is complex decongestive therapy (CDT), which primarily involves a specialized gentle type of therapeutic massage known as manual lymphatic drainage (MLD), followed by exercise, compression therapy, and skin care (1, 2, 13, 14). Randomized controlled trials (RCTs) have demonstrated statistically significant improvements in the incidence of LE and pain following MLD in patients with breast cancer (*p* = .02) (15). Pneumatic compression therapy (PCT) is part of CDT and usually involves a garment inflated by a pump over the affected limb to mimic MLD in the home setting (16). Multiple studies have demonstrated that significant edema reduction occurred with PCT (16–19).

Over the past decade, improved techniques in lymphatic imaging have demonstrated lymphatic contractile function following LE treatment. Near-infrared fluorescence lymphatic imaging (NIRFLI) visualizes lymphatic function and movement in real-time, by tracing the flow of intradermal injections of indocyanine green (ICG) administered to relevant anatomic sites (6, 16, 20–24). NIRFLI has shown that lymphatic function improves following MLD (21) and PCT (16, 23, 24). However, while improved lymphatic pumping, measured by increased pulsatile frequencies of lymphatic vessels, has been consistently observed with NIRFLI, lymphatic mapping of drainage patterns is highly heterogeneous and variable from one patient to the next, even among the same affected anatomic regions (25). Lymphatic vessels can both converge into one and split into multiples at the same site, and their drainage pathways and rates vary enormously.

A novel PCT medical device is now available over the counter for home use that involves a 16-chamber mat on which patients lie down supine to receive automated MLD therapy (AMLDT). The US Food and Drug Administration classifies it as a Class II medical device indicated for temporary pain reduction in people with good health, excluding pregnancy, heart/vascular problems, acute pulmonary edema, infections, injury at/near the application site, where increased venous/lymphatic return is undesirable, spinal cord/back injuries, and bronchial asthma. Herein, 4 case reports describe a patient with traumatic CRPS and LE, a healthy patient, a breast cancer survivor with chronic pain, and a patient with a history of abdominal surgery. They serve as proof of concept to assess how AMLDT impacts lymphatic function and also demonstrate their unique lymphatic drainage patterns. Each case underwent NIRFLI while receiving a single session of AMLDT to determine whether AMLDT improved lymphatic pumping, specifically pulsatile frequency. Secondarily, patient feedback on AMLDT and its effect on pain was assessed to understand patient perspective.

## 2 Case description

All 4 patients presented at a university outpatient clinic in August 2023 and provided their written informed consent. The Committee for the Protection of Human Subjects approved the study protocol (HSC-MS-23-0093) on June 22, 2023. In all cases, vital signs were normal at time of presentation.

### 2.1 Case 1

A 58-year-old, White male patient presented with CRPS and stage 1 LE on the right calf and foot. He had no relevant family history and had been taking diosmin/hesperidin and Vitamin C, D3, and B-complex supplements for 2 years. He was in a motor vehicle accident in 1997, resulting in a complex right ankle and tibia/fibula fracture and compartment syndrome. He underwent 16 surgeries over a 3-week period, including external fixator placement, 4-compartment fasciotomy, debridement, skin grafting, and bone grafting. The CRPS was initially managed with lumbar sympathectomies, pain medications, and acupuncture therapy. At the time of presentation, he used physical exercise and compression stocking to manage the CRPS symptoms.

### 2.2 Case 2

A 54-year-old, healthy, White Hispanic male patient presented with no relevant history; he had no chronic pain or LE. He underwent right Achilles tendon repair in 1985 and abdominal hernia repair in 2008. He underwent a left partial thyroidectomy in 2005 and took levothyroxine for 23 years.

### 2.3 Case 3

A 71-year-old, White female, 30-year breast cancer survivor presented with RA, chronic back pain due to degenerative joint disease, and mild scoliosis. Medications included lisinopril, a statin, levothyroxine, antidepressants, pantoprazole, hydroxychloroquine and supplemental vitamins, turmeric, and probiotics. Chronic pain was managed with gabapentin, naproxen, and tizanidine. She underwent a lumpectomy of the left breast in 1992 and had a history of non-Hodgkins lymphoma from 2015. She had titanium rods in both femurs due to previous fractures in 2007, with additional right femur hardware revisions in 2014 and 2016. She sustained multiple fractures to her arm in 2017 and 2018. Her RA had been managed monthly for the past 20 years with an immunomodulating drug.

### 2.4 Case 4

A 64-year-old, White female presented with a history of cosmetic abdominoplasty and brachioplasty (3 years prior). She had mild hypertension, managed with daily losartan, Hashimoto's thyroiditis, and a history of obesity, which resolved following Roux e Y gastric bypass 18 years prior. Medications included thyroid supplementation, postmenopausal estrogen replacement, and botox injections to the scalp every 3 months for migraines.

## 3 NIRFLI procedure

Each case underwent a NIRFLI session with concurrent AMLDT. NIRFLI assessed lymphatic flow at baseline with the patient in a supine and prone position, during 1 h of AMLDT with the patient in a supine position, and 30–60 min posttreatment with the patient in a supine and prone position. At the start of the imaging session, patients reported pain using a Visual Analogue Scale (VAS, based on a score between 0 and 10, with 0 having no pain and 10 having the most pain). Each patient put on a “bikini” bottom, and women wore a bikini top, to afford maximal visualization of dermal lymphatic regions.

**Figure 1:**
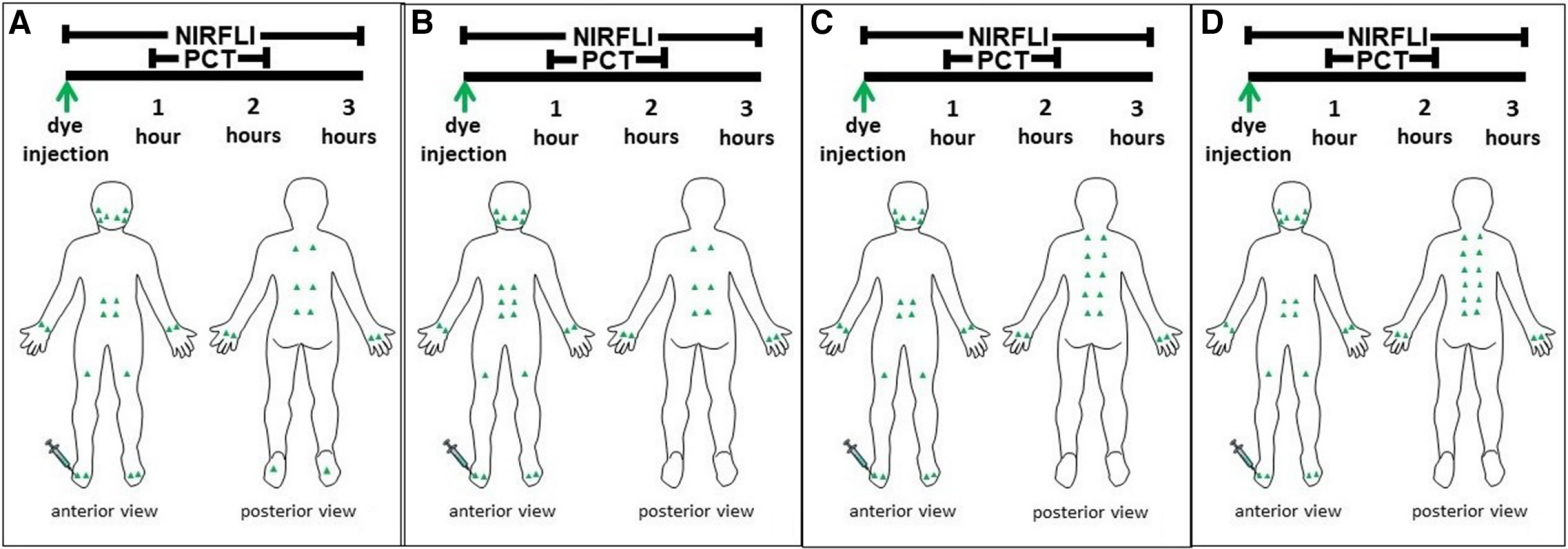
Green triangles represent the indocyanine green injection sites for each case on both the front and back of their body. Near-infrared fluorescence lymphatic imaging (NIRFLI) was obtained for up to 3 h. The first hour captured lymphatic flow immediately before treatment with the automated manual lymphatic drainage mat. The second hour captured lymphatic flow while each patient underwent 1-hour of concurrent pneumatic compression therapy (PCT) with the automated manual lymphatic drainage mat. The final 30 to 60 min captured posttreatment lymphatic flow. The injection protocol for Case 1, a 58-year-old male patient with complex regional pain syndrome and stage 1 lymphedema (LE) in his right foot and calf is depicted in 1A. The protocol for Case 2, a 52-year-old, healthy male patient with no relevant history of LE is shown in 1B. The protocol for Case 3, a 71-year-old breast cancer survivor with chronic pain from rheumatoid arthritis and degenerative joint disease and a history of LE is shown in 1C. The protocol for Case 4, a 64-year old female patient with a history of abdominoplasty and brachioplasty that caused prior LE is shown in 1D.

The ICG (25 mg vial) was reconstituted with 10 ml sterile water and further diluted in sterile saline immediately prior to administration. The final concentration of 0.32 mM provided 25 μg ICG/injection, with each injection of 0.1 cc made at the anatomical injection sites shown in Figure 1. Tables 1, 2 also describe the anatomic injection sites of each case. Alcohol wipes were used to clean each injection site before intradermal injection. Each patient received 32–36 injections. NIRFLI was accomplished using a custom system consisting of a Gen III military-grade intensifier coupled to a frame transfer, 16-bit camera with 200 ms exposure times (20). Initial NIRFLI imaging lasted for 60 min to assess the drainage of ICG laden lymph from each injection site toward regional nodal basins. Vital signs of blood pressure, pulse rate, respiratory rate, temperature, and redness or inflammation at the injection site were monitored and recorded at baseline (before ICG injection) and 15 and 30 min after injection. NIRFLI imaging continued during the 1-h AMLDT session. After AMLDT, imaging continued for another 30–60 min. Patients reported any posttreatment pain and provided their feedback on the AMLDT device.

**Table 1:** Anterior pulsatile frequencies (pulses/min) captured by near-infrared fluorescence lymphatic imaging before, during, and after treatment (txt) with automated manual lymphatic drainage therapy.

Anatomic injection site	Case 1, pulses/min			Case 2, pulses/min			Case 3, pulses/min			Case 4, pulses/min		
	Pre-txt	Inter-txt	Post-txt	Pre-txt	Inter-txt	Post-txt	Pre-txt	Inter-txt	Post-txt	Pre-txt	Inter-txt	Post-txt
Neck												
Right							ND	ND	0.68			
Left							ND	ND	0.68			
Arms												
Right	1.45, 0,73	N/A	0.96	1.84	2.16	5.82	1.68	ND	ND	1.27	2.38	4.41
Left	1.43, 0.71	N/A	0.96	1.54	2.17	1.56	1.68	ND	ND	1.27	2.46	3.31
Umbilical region												
Superior umbilical-to-axilla												
Right				0.59, 1.09	0.51, 0.66	0						
Left				2.51	1.98	0.88						
Middle umbilical-to-axilla												
Right	NV	1.08	NV				3.55	0.41	1.70	0	0	0
Left	0.69	1.08	0.90				2.72	0	1.70	0	0	0
Middle umbilical-to-inguinal												
Right				1.42	0.15	0						
Left				1.54	0.59	0						
Inferior umbilical-to-inguinal												
Right	0.71	0.83, 0.83	STR	1.51	0.51	0	0	0	ND	0	0	0
Left	0.71	0.91	0.47, 1.07	0	0	0	0	0	ND	0	0.30	0
Anterior legs												
Lateral thigh-to-inguinal												
Right	1.88	NV	NV	0.17	0	0.29	0	NV	ND	0.55	0.44	NV
Left	0.59	NV	ND	0.50	0	0.48	0	NV	ND	0.82	0.44	NV
Medial thigh-to-inguinal												
Right				0.33	0.38	0.48	0	NV	ND	ND	ND	ND
Left				0.33	0.25	1.25	STR	1.25	ND	ND	ND	ND
Inferior												
Right	0.53	0.96	0.75	1.04	0.75	1.79	1.05	1.22	1.23	0.85	0.65	0.72
Left	0.72	0.72	0.69	0.45	0.87	0.89	1.62	0.77	0.89	0.85	0.39	0.72

Injection sites are listed in top down order as depicted in Figure 1. Multiple values per cell represent multiple vessels emanating from injection site. Empty cells indicate data were not applicable. ND, Not done; data not obtained, given the time constraints; NV, not visible; STR, streaming.

**Table 2:** Posterior pulsatile frequencies (pulses/min) captured by near-infrared fluorescence lymphatic imaging before, during, and after treatment (txt) with automated manual lymphatic drainage therapy on the back of the body.

Anatomic injection site	Case 1, pulses/min			Case 2, pulses/min			Case 3, pulses/min			Case 4, pulses/min		
	Pre-txt	Inter-txt	Post-txt	Pre-txt	Inter-txt	Post-txt	Pre-txt	Inter-txt	Post-txt	Pre-txt	Inter-txt	Post-txt
Back												
1st superior back												
Right	1.20, 1.20	ND	1.06, 0.85	0.98	ND	0.50	0	ND	0	0.81	ND	NV
Left	1.55	ND	0.53	1.28	ND	0.92	0	ND	0	0.95	ND	1.80, 0.78
2nd superior back												
Right							0	ND	0			
Left							0	ND	0			
Posterior thoracic area												
Right	1.08	ND	0.85	0.39	ND	1.17	0.46	ND	0.60			
Left	1.20	ND	0.95	0.69	ND	0.42	1.94	ND	1.07			
1st lumbar area												
Right	STR	ND	0.53	1.28	ND	0.42	1.08	ND	0.60	0.68	ND	1.48
Left	1.55, 0.84	ND	1.59, 0.84	1.08	ND	0.50	0	ND	NV	0.68	ND	0.87
2nd lumbar area												
Right							0.54	ND	0.36	1.63	ND	1.39
Left							0	ND	NV	0.95	ND	0.61
3rd lumbar area												
Right										0.68	ND	0.52
Left										0.81	ND	1.04
4th lumbar area												
Right										NV	ND	NV
Left										1.08	ND	0.61
Posterior legs												
Right	ND	ND	0.66									
Left	ND	ND	0.44									

Injection sites are listed in top down order as depicted in Figure 1. Multiple values per cell represent multiple vessels emanating from injection site. Empty cells indicate data were not applicable. ND, Not done; data not obtained, given the time constraints; NV, not visible; STR, streaming.

### 3.1 NIRLFI analysis

A trained imager from the molecular imaging center analyzed NIRFLI images using ImageJ (US National Institutes of Health). Movies were generated from sequences of NIRFLI and depicted movement of lymphatic vessels and lymph nodes. A region of interest (ROI) was chosen along each lymphatic vessel, and the increasing/decreasing fluorescence intensity associated with each pulse observed as it moved through the ROI in successive movie frames. The timelapse was determined from the time stamps of the first and last images in each movie. The number of pulses observed in each vessel ROI was divided by the corresponding timelapse to render pulsing frequency (pulses/min) for each vessel, which is an outcome measure of lymphatic function (20). The investigators only had a 2-day period to evaluate all 4 cases, when at least 4 days were needed to collect complete timepoint data. Given these time constraints due to laboratory availability, it was necessary to omit some timepoints from data collection and analysis. Thus, the investigators focused on the back and trunk, as well as areas of interest specific to each case (such as the first case's foot with CRPS). All relevant data were collected in an Excel spreadsheet.

## 4 AMLDT procedure

The AMLDT system (Neuroglide, Eva Medtec, Bloomington, MN) offers 3 treatment focus zones (Upper and Lower Back/Neck, Upper Back/Neck, and Lower Back) and 3 pressure settings (mild, medium, and intense). The back/neck pad has 16 air channels precisely timed to produce a sequential inflation/deflation pressure cycle, mimicking the stretch and release action provided by therapeutic MLD-like massage to manipulate soft tissue, relieve pain, and increase circulation. The AMLDT system is intended for use on a flat, firm surface at home.

Each patient lay supine on the pad with their heads centered on the supportive pillow section, ensuring that there were no gaps between their neck and the pad. They received 1 h of intense AMLDT to the upper and lower back/neck.

## 5 Results/cases 1–4

**Figure 2:**
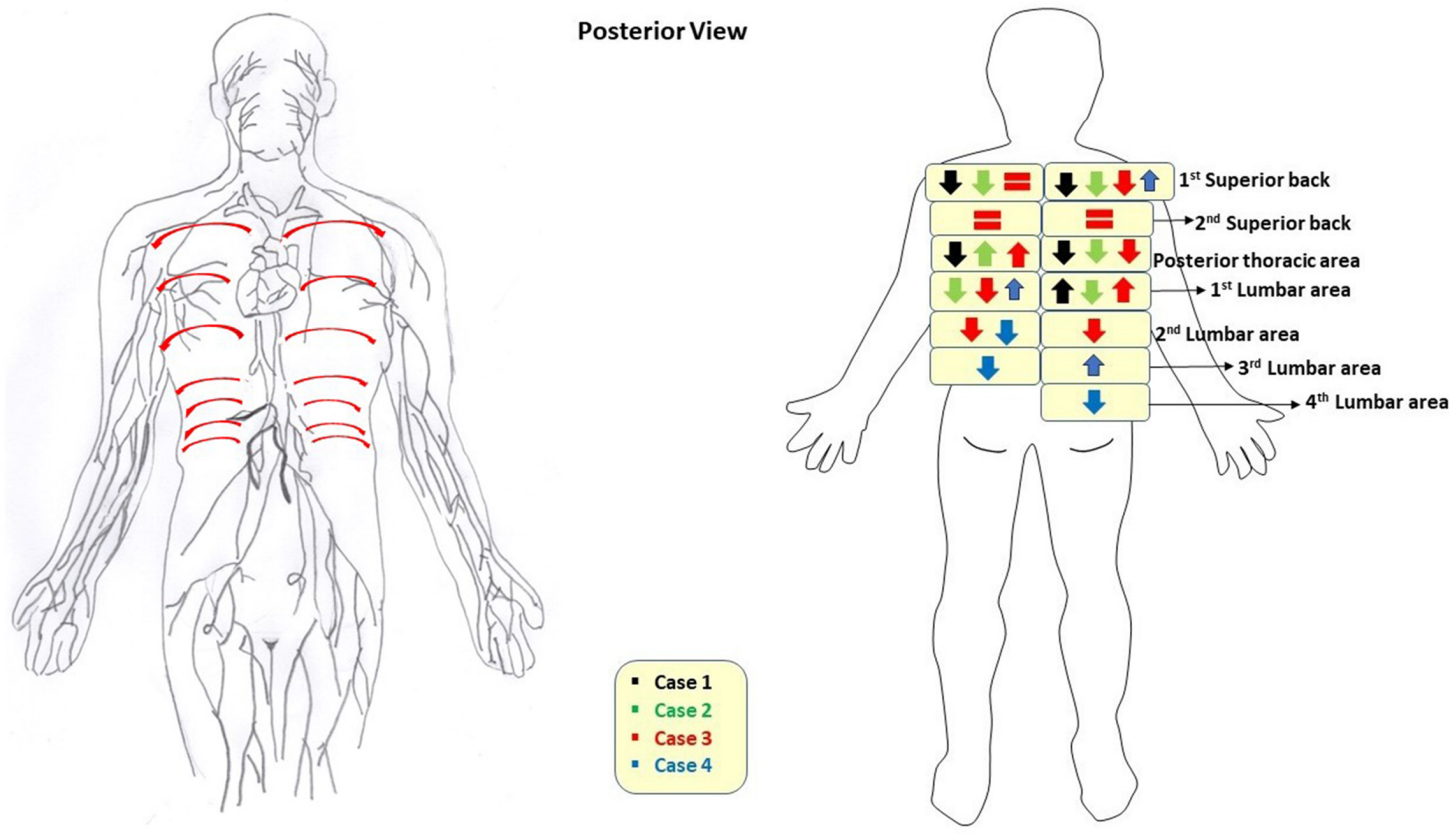
On the left, an avatar of the “expected” lymph drainage on the back of the body is pictured, with red arrows indicating directional flow from all injection sites summarized in Figure 1. On the right, an avatar of directional changes of pulsatile frequencies measured by near-infrared fluorescence lymphatic imaging on the back after treatment with automated lymphatic manual drainage therapy is pictured. Directional changes show the difference from pretreatment to posttreatment, when data were available. No intertreatment data were available. If there was no change in data (i.e., no increase or decrease in pulses/min posttreatment), then an equal sign is shown.

All 4 cases completed the NIRFLI and AMLDT session without complications or adverse events. Tables 1, 2 report the lymphatic pulsing frequencies for visualized vessels draining at each injection site (listed in top down order as depicted in Figure 1) 1 h before AMLDT, during treatment, and 30–60 min posttreatment. When multiple vessels drained at an injection site, a pulsing frequency for each is reported. AMLDT appeared to alter lymphatic contractility; both increased and decreased pulsing frequencies were observed, including in healthy, nonaffected limbs (Case 2). Pulsing frequencies were very heterogeneous from one case to the next and also varied within anatomic regions of the same patient. Figure 2 shows avatar examples of the heterogeneity of the directional changes in pulsing frequencies on the backs of each case.

In Case 1, NIRFLI demonstrated lymphatic dysfunction with dermal backflow, in which lymph moves into the lymphatic capillary bed instead of unidirectionally into lymphatic collector vessels, and tortuous vessels in the right upper lateral shin and foot. Generally, injection sites on the arms, anterior abdomen, and anterior legs of the front of his body saw increases in pulsatile frequency during AMLDT and posttreatment, compared to baseline values (Table 1). Most notably, in the CRPS-affected right leg, the pulsatile frequency nearly doubled during AMLDT from 0.53 pulses/min to 0.96 pulses/min and remained higher than baseline during posttreatment NIRFLI (0.75 pulses/min). At the umbilical region, drainage patterns varied, with the inferior umbilical injection sites and possibly the left superior injection site draining to the inguinal region, while the right superior umbilical injection site drained towards the axilla. On his back, the injections at the thoracic level drained inferiorly and laterally and then traveled anteriorly towards axilla. Supplementary Videos 1A and 1B depict NIRFLI before and after AMLDT on his back, where lymphatic collectors traveled from medial-to-lateral in a dermatomal pattern and pulsatile frequencies decreased after treatment, except in the 2nd to 4th lumbar areas where no data was obtained (Table 2).

Case 2 (healthy patient) had increases in pulsatile frequencies on his arms, thighs, and lower legs in response to treatment (Table 1). Most notably, pulsatile frequency more than tripled in his right arm from 1.84 pulses/min before AMLDT to 5.82 after treatment. On the back, however, pulsatile frequencies generally decreased after treatment, except for his right lumbar area, where the rate tripled from 0.39 pulses/min before treatment to 1.17 pulses/min after treatment (Table 2).

Case 3 notably had a lack of pulsing or lack of data at most injection sites at the umbilical region, legs, and back, and (particularly) near the anterior and posterior neck (Tables 1, 2). One small, unusual vessel was observed on her left arm, which previously experienced a fracture and was the side corresponding to her breast cancer. Her middle umbilical-to-axilla sites saw sharp decreases in pulsatile frequencies after AMLDT, while sites on her right leg and right posterior thoracic area increased.

NIRFLI did not show pulsing on the umbilical sites of Case 4, expect for one tortuous vessel that was captured on the left lower umbilical-to-inguinal site during AMLDT, with a low rate of 0.30 pulses/min (Table 1). Pulsing frequencies notably increased on both her arms during and after treatment, with the posttreatment rates 3.5 higher in her right arm and 2.6 times higher in her left arm compared to baseline. In her legs, however, pulsatile frequencies decreased. On her back, rate increases were observed at the most superior injection at the posterior thorax on the right, both superior lumbar sites, and the left inferior lumbar site (Table 2). All other back sites demonstrated decreased rates after AMLDT, or data was unavailable.

From the patients' subjective perspective, AMLDT was reported as soothing and/or relaxing. Decreased pain was reported in the 3 cases that reported pretreatment pain. The VAS scores for Case 1 decreased from 5 to 2 after treatment, in Case 3 from 2 to 0, and in Case 4 from 4 to 0. Case 2 did not report any pain during this study.

## 6 Discussion

We describe 4 very heterogenous cases with and without LE or risk of LE, all of whom demonstrated altered lymphatic movement (as shown by NIRFLI) after 1 h of AMLDT. AMLDT provided pain relief to those affected with chronic pain. AMLDT is intended for pain relief; thus, the patient feedback in these case reports is not surprising, but a further discussion of the association of the lymphatic system with chronic pain is warranted, especially in the context of Case 1. A companion paper will explore the effect of AMLDT on his CRPS and LE in greater detail.

Pulsatile frequences increased and decreased in all cases, suggesting changes in lymphatic contractility trends, and findings were not necessarily consistent across the anatomic region of the same patient (Tables 1, 2). The decreased pulsatile frequencies generally observed on the back (Table 2, Figure 2), and the finding that back lymphatic collectors travel from medial to lateral along dermatomal patterns (Supplementary Videos 1A and 1B) was clearly demonstrated, but it is not unusual for unexpected lymphatic movements and drainage patterns to occur in both healthy-appearing and unaffected anatomic regions (22, 25). High-quality lymphoscintigraphy has shown that in over 3,000 patients with melanomas, the trunk of the body frequently demonstrated drainage across the body's midline (25), and superficial lymph flow moving laterally and a deep lymph flow moving medially has been reported in the back (26). With NIRLFI, improved imaging technology now captures real-time lymphatic movement, and our understanding of lymphatic movement is undergoing a paradigm shift from defining “normal” flow in healthy patients towards the observation that every patient has their own unique watershed zones that may not be static over time. This evolving evidence base will allow for more targeted therapeutic outcomes and advance personalized medicine.

The unexpected, decreased pulsatile frequencies observed in the back do not appear to be a limitation of the AMLDT pumping technique (Figure 2). What is unique to the AMLDT mat used in this study, compared to other MLD/PCT devices, is that the treatment not only has an effect upon limb lymphatic contractility, but also along the back, with the patient resting in a supine position, rather than seated (16–19). We speculate that the relaxation induced by AMLDT, coupled with the gravity exertion on the back, resulted in decreased pulsatile frequencies, similar to a lower resting heart rate. Both extrinsic and intrinsic forces are responsible for lymphatic movement, with the latter driven by lymph vessel distension. The lymphangion, the pumping unit bounded by valves located along collector lymphatic vessels, is known to function similarly to the cardiac pump in the heart, by increasing intrinsic contractility to adapt to output pressure elevation in response to increased pressure and/or gravitational load, producing a systolic and diastolic phase (27–31). This intrinsic pumping mechanism varies contractility rates and lymph flow to respond to environmental factors and pressure gradients. Jamalian et al. (29) supported Guyton's subatmospheric theory, which hypothesizes that a lymphatic contraction produces a suction effect (in the presence of competent lymphatic valves) upon relaxing of decreased pressure downstream, opening the inlet valve and allowing for the filling of lymphatic fluid due to the low upstream pressure from the previous section of vessel (32, 33). Therefore, the relaxation of the collecting vessel following contraction is essential to drawing fluid from the interstitium. Since our patients received AMLDT to the back, it is possible that the gravitational load moved a sufficient volume of fluid during treatment, decreasing output pressure and resulting in decreased contractility and pulsatile frequencies. Additionally, mapping of lymphatic drainage following MLD applied to improve LE of the upper back has shown that MLD essentially finds an alternative, efficient route to evacuate accumulated lymph interstitial fluid to an area not affected with LE (34). These alternative lymphatic drainage pathways are rerouted, as needed, on an individual basis, further explaining the heterogeneity of patient data. Further study of AMLDT on the back of the body is suggested to confirm these findings.

Only 4, noncomparative cases are reported in this paper, and so statistical analysis was not performed. While the cases represent different health states and LE status and may not be generalizable to the general population, they nonetheless support the heterogeneous anatomy and function of the lymphatic system that previously has been reported in the literature (22, 25) and show the change in lymphatic contractility from before AMLDT (baseline) to posttreatment. Each patient only received 1 treatment session of AMLDT, and so, the long-term effect of AMLDT is unknown. In a study of 10 survivors of head and neck cancer with LE, NIRFLI demonstrated that daily use of advanced PCT for 2 weeks improved lymphatic function in all patients and eliminated or reduced dermal backflow in 6 of the 8 patients who had had backflow (23). There remains a need to better characterize the propulsion frequency over time. Future comparative studies could potentially control for time variations by imaging controls over the same timeframe, with a longer follow-up time, and in the same positions but without AMDLT. This proof-of-concept study suggests that, in addition to reducing pain, AMLDT appears to impact lymphatic contractility of the lower and upper extremities, umbilical region/trunk, and back. The resting, supine position required for AMLDT likely decreased contractility rates and lowered pulsatile frequency during and after treatment, further supporting that lymphangion pumps respond to intrinsic and extrinsic forces similar to the cardiovascular system and have varying contractile rates in response to changing environmental pressures. Further research on the effect of AMLDT on the back and on patients with LE and chronic pain is warranted.

## Data Availability

The original contributions presented in the study are included in the article/**Supplementary Material**, further inquiries can be directed to the corresponding author.

## Supplementary material

Supplemental Video 1A A video showing near-infrared fluorescence lymphatic imaging of the back before automated manual lymphatic drainage therapy of Case 1, a 58-year-old, White male patient with complex regional pain syndrome and stage 1 lymphedema on the right calf. Brightness and contrast have been adjusted to help visualize dimmer fluorescent signals. The diagram in the upper left corner in the first few frames illustrates the location of the indocyanine green injection sites (x) and the imaging field of view (red circle) in the video. The yellow arrow in some frames highlights the movement of an example propulsion event from the upper injection site towards the left axilla. Lymphatic collectors are observed to travel medial to lateral on the back. For the left side of the back, at baseline, pulsatile frequencies were 1.55 for the superior back indocyanine green injection site, 1.20 for the posterior thoracic area site, and 1.55 and 0.84 for the 2 different vessels emanating from the lumbar area injection site. For the right side of the back, baseline pulsatile frequencies were 1.20 and 1.20 for 2 different vessels emanating from the superior back injection site, 1.08 for the thoracic area site, and undetermined (streaming) for the lumbar area site.

Supplemental Video 1B A video showing near-infrared fluorescence lymphatic imaging of the back of Case 1 after 1 h of automated manual lymphatic drainage therapy. Brightness and contrast have been adjusted to help visualize dimmer fluorescent signals. The diagram in the upper left corner in the first few frames illustrates the location of the indocyanine green injection sites (x) and the imaging field of view (red circle) in the video. The yellow arrow in some frames highlights the movement of an example propulsion event from the upper injection site towards the left axilla. Pulsatile frequencies decreased following treatment. On the left side of the back, pulsatile frequencies were 0.53, 0.95, and 1.59 and 1.16 for the respective superior back, thoracic area, and lumbar area indocyanine green injection sites. On the right side of the back, the respective pulsatile frequencies were 1.06 and 0.85, 0.85, and 0.53.

## References

[1] de Sire A Losco L Lippi L Spadoni D Kaciulyte J Sert G Surgical treatment and rehabilitation strategies for upper and lower extremity lymphedema: a comprehensive review. Medicina (Kaunas). (2022) 58(7):954. 10.3390/medicina58070954 35888673 PMC9324426

[2] Brix B Sery O Onorato A Ure C Roessler A Goswami N. Biology of lymphedema. Biology (Basel). (2021) 10(4):261. 10.3390/biology10040261 33806183 PMC8065876

[3] Breslin JW Yang Y Scallan JP Sweat RS Adderley SP Murfee WL. Lymphatic vessel network structure and physiology. Compr Physiol. (2018) 9(1):207–99. 10.1002/cphy.c180015 30549020 PMC6459625

[4] Schwager S Detmar M. Inflammation and lymphatic function. Front Immunol. (2019) 10:308. 10.3389/fimmu.2019.00308 30863410 PMC6399417

[5] Tuckey B Srbely J Rigney G Vythilingam M Shah J. Impaired lymphatic drainage and interstitial inflammatory stasis in chronic musculoskeletal and idiopathic pain syndromes: exploring a novel mechanism. Front Pain Res (Lausanne). (2021) 2:691740. 10.3389/fpain.2021.691740 35295453 PMC8915610

[6] Aldrich MB Sevick-Muraca EM. Cytokines are systemic effectors of lymphatic function in acute inflammation. Cytokine. (2013) 64(1):362–9. 10.1016/j.cyto.2013.05.015 23764549 PMC3771384

[7] Lopez M Roberson ML Strassle PD Ogunleye A. Epidemiology of lymphedema-related admissions in the United States: 2012–2017. Surg Oncol. (2020) 35:249–53. 10.1016/j.suronc.2020.09.005 32932222

[8] Cormier JN Askew RL Mungovan KS Xing Y Ross MI Armer JM. Lymphedema beyond breast cancer: a systematic review and meta-analysis of cancer-related secondary lymphedema. Cancer. (2010) 116(22):5138–49. 10.1002/cncr.25458 20665892

[9] Ren Y Kebede MA Ogunleye AA Emerson MA Evenson KR Carey LA Burden of lymphedema in long-term breast cancer survivors by race and age. Cancer. (2022) 128(23):4119–28. 10.1002/cncr.34489 36223240 PMC9879608

[10] Jayson MI Cavill I Barks JS. Lymphatic clearance rates in rheumatoid arthritis. Ann Rheum Dis. (1971) 30:638–9. 10.1136/ard.30.6.638 5130143 PMC1005842

[11] Sahbaie P Li WW Guo TZ Shi XY Kingery WS Clark JD. Autonomic regulation of nociceptive and immunologic changes in a mouse model of complex regional pain syndrome. J Pain. (2022) 23(3):472–6. 10.1016/j.jpain.2021.09.009 34699985 PMC8920776

[12] Howarth D Burstal R Hayes C Lan L Lantry G. Autonomic regulation of lymphatic flow in the lower extremity demonstrated on lymphoscintigraphy in patients with reflex sympathetic dystrophy. Clin Nucl Med. (1999) 24(6):383–7. 10.1097/00003072-199906000-00001 10361930

[13] Lurie F Malgor RD Carman T Dean SM Iafrati MD Khilnani NM The American venous forum, American vein and lymphatic society and the society for vascular medicine expert opinion consensus on lymphedema diagnosis and treatment. Phlebology. (2022) 37(4):252–6. 10.1177/02683555211053532 35258350 PMC9069652

[14] Executive Committee of the International Society of Lymphology. The diagnosis and treatment of peripheral lymphedema: 2020 consensus document of the international society of lymphology. Lymphology. (2020) 53(1):3–19. 10.2458/lymph.4649 32521126

[15] Lin Y Yang Y Zhang X Li W Li H Mu D. Manual lymphatic drainage for breast cancer-related lymphedema: a systematic review and meta-analysis of randomized controlled trials. Clin Breast Cancer. (2022) 22(5):e664–73. 10.1016/j.clbc.2022.01.013 35370085

[16] Aldrich MB Gross D Morrow JR Fife CE Rasmussen JC. Effect of pneumatic compression therapy on lymph movement in lymphedema-affected extremities, as assessed by near-infrared fluorescence lymphatic imaging. J Innov Opt Health Sci. (2017) 10(2):1650049. 10.1142/S1793545816500498 29104671 PMC5665410

[17] Pappas CJ O'Donnell TF Jr. Long-term results of compression treatment for lymphedema. J Vasc Surg. (1992) 16(4):555–64. 10.1067/mva.1992.39929 1404677

[18] Richmand DM Jr OT Zelikovski A. Sequential pneumatic compression for lymphedema. A controlled trial. Arch Surg. (1985) 120(10):1116–9. 10.1001/archsurg.1985.01390340014002 4038053

[19] BadgerCPrestonNSeersKMortimerP. Physical therapies for reducing and controlling lymphoedema of the limbs. Cochrane Database Syst Rev. (2004) 4:CD003141. 10.1002/14651858.CD003141.pub215495042

[20] Rasmussen JC Tan I-C Marshall MV Adams KE Kwon S Fife CE Human lymphatic architecture and dynamic transport imaged using near-infrared fluorescence. Transl Oncol. (2010) 3:362–72. 10.1593/tlo.10190 21151475 PMC3000461

[21] Tan IC Maus EA Rasmussen JC Marshall MV Adams KE Fife CE Assessment of lymphatic contractile function after manual lymphatic drainage using near-infrared fluorescence imaging. Arch Phys Med Rehabil. (2011) 92(5):756–64.e1. 10.1016/j.apmr.2010.12.027 21530723 PMC3109491

[22] Rasmussen JC Zhu B Morrow JR Aldrich MB Sahihi A Harlin SA Degradation of lymphatic anatomy and function in early venous insufficiency. J Vasc Surg Venous Lymphat Disord. (2021) 9(3):720–30.e2. 10.1016/j.jvsv.2020.09.007 32977070 PMC7982349

[23] Gutierrez C Karni RJ Naqvi S Aldrich MB Zhu B Morrow JR Head and neck lymphedema: treatment response to single and multiple sessions of advanced pneumatic compression therapy. Otolaryngol Head Neck Surg. (2019) 160(4):622–6. 10.1177/0194599818823180 30694720

[24] Rasmussen JC Aldrich MB Tan IC Darne C Zhu B O’Donnell TF Lymphatic transport in patients with chronic venous insufficiency and venous leg ulcers following sequential pneumatic compression. J Vasc Surg Venous Lymphat Disord. (2016) 4(1):9–17. 10.1016/j.jvsv.2015.06.001 26946890 PMC4782606

[25] Uren RF Howman-Giles R Thompson JF. Patterns of lymphatic drainage from the skin in patients with melanoma. J Nucl Med. (2003) 44(4):570–82. 12679402

[26] Rickenbacher J Landolt AM Theiler K Scheier H Siegfried J Wagenhaüser FJ Outline of the lymphatic system of the back. In: Applied Anatomy of the Back. Berlin, Heidelberg: Springer (1985). p. 113–7. 10.1007/978-3-662-05791-97

[27] Davis MJ Scallan JP Wolpers JH Muthuchamy M Gashev AA Zawieja DC. Intrinsic increase in lymphangion muscle contractility in response to elevated afterload. Am J Physiol Heart Circ Physiol. (2012) 303(7):H795–808. 10.1152/ajpheart.01097.2011 22886407 PMC3469705

[28] Scallan JP Zawieja SD Castorena-Gonzalez JA Davis MJ. Lymphatic pumping: mechanics, mechanisms and malfunction. J Physiol. (2016) 594(20):5749–68. 10.1113/JP272088 27219461 PMC5063934

[29] Jamalian S Jafarnejad M Zawieja SD Bertram CD Gashev AA Zawieja DC Demonstration and analysis of the suction effect for pumping lymph from tissue beds at subatmospheric pressure. Sci Rep. (2017) 7(1):12080. 10.1038/s41598-017-11599-x 28935890 PMC5608746

[30] Bertram CD Macaskill C Davis MJ Moore JE Jr. Contraction of collecting lymphatics: organization of pressure-dependent rate for multiple lymphangions. Biomech Model Mechanobiol. (2018) 17(5):1513–32. 10.1007/s10237-018-1042-7 29948540 PMC6139274

[31] Solari E Marcozzi C Negrini D Moriondo A. Lymphatic vessels and their surroundings: how local physical factors affect lymph flow. Biology (Basel). (2020) 9(12):463. 10.3390/biology9120463 33322476 PMC7763507

[32] Guyton AC Coleman TG. Regulation on interstitial fluid volume and pressure. Ann N Y Acad Sci. (1968) 150(3):537–47. 10.1111/j.1749-6632.1968.tb14705.x 5248766

[33] Guyton AC Granger HJ Taylor AE. Interstitial fluid pressure. Physiol Rev. (1971) 51:527–63. 10.1152/physrev.1971.51.3.527 4950077

[34] Chikly B Quaghebeur J Witryol W. A controlled comparison between manual lymphatic mapping (MLM) of plantar lymph flow and standard physiologic maps using lymph drainage therapy (LDT)/osteopathic lymphatic technique (OLT). J Yoga Phys Ther. (2014) 4:4. 10.4172/2157-7595.1000173

