# Supplementary material for: Case Report: The effect of automated manual lymphatic drainage therapy on lymphatic contractility in 4 distinct cases
Source: Front Med Technol. 2024 Jul 17;6:1397561. doi: 10.3389/fmedt.2024.1397561 (PMC11292613; doi:10.3389/fmedt.2024.1397561)
Supplement: Supplementary file 3 [file Table1.docx]

Table 1. Anterior pulsatile frequencies (pulses/minute) captured by near-infrared fluorescence lymphatic imaging before, during, and after treatment (txt) with automated manual lymphatic drainage therapy. Injection sites are listed in top down order as depicted in Figure 1. Multiple values per cell represent multiple vessels emanating from injection site. Empty cells indicate data were not applicable.

| **Anatomic Injection Site** | **Case 1, pulses/min** | | | **Case 2, pulses/min** | | | **Case 3, pulses/min** | | | **Case 4, pulses/min** | | |
| --- | --- | --- | --- | --- | --- | --- | --- | --- | --- | --- | --- | --- |
|  | Pre-txt | Inter-txt | Post-txt | Pre-txt | Inter-txt | Post-txt | Pre-txt | Inter-txt | Post-txt | Pre-txt | Inter-txt | Post-txt |
| Neck |  |  |  |  |  |  |  |  |  |  |  |  |
| Right |  |  |  |  |  |  | ND | ND | 0.68 |  |  |  |
| Left |  |  |  |  |  |  | ND | ND | 0.68 |  |  |  |
| Arms |  |  |  |  |  |  |  |  |  |  |  |  |
| Right | 1.45, 0,73 | N/A | 0.96 | 1.84 | 2.16 | 5.82 | 1.68 | ND | ND | 1.27 | 2.38 | 4.41 |
| Left | 1.43, 0.71 | N/A | 0.96 | 1.54 | 2.17 | 1.56 | 1.68 | ND | ND | 1.27 | 2.46 | 3.31 |
| Umbilical region |  |  |  |  |  |  |  |  |  |  |  |  |
| Superior umbilical-to-axilla |  |  |  |  |  |  |  |  |  |  |  |  |
| Right |  |  |  | 0.59, 1.09 | 0.51, 0.66 | 0 |  |  |  |  |  |  |
| Left |  |  |  | 2.51 | 1.98 | 0.88 |  |  |  |  |  |  |
| Middle umbilical-to-axilla |  |  |  |  |  |  |  |  |  |  |  |  |
| Right | NV | 1.08 | NV |  |  |  | 3.55 | 0.41 | 1.70 | 0 | 0 | 0 |
| Left | 0.69 | 1.08 | 0.90 |  |  |  | 2.72 | 0 | 1.70 | 0 | 0 | 0 |
| Middle umbilical-to-inguinal |  |  |  |  |  |  |  |  |  |  |  |  |
| Right |  |  |  | 1.42 | 0.15 | 0 |  |  |  |  |  |  |
| Left |  |  |  | 1.54 | 0.59 | 0 |  |  |  |  |  |  |
| Inferior umbilical-to-inguinal |  |  |  |  |  |  |  |  |  |  |  |  |
| Right | 0.71 | 0.83, 0.83 | STR | 1.51 | 0.51 | 0 | 0 | 0 | ND | 0 | 0 | 0 |
| Left | 0.71 | 0.91 | 0.47, 1.07 | 0 | 0 | 0 | 0 | 0 | ND | 0 | 0.30 | 0 |
| Anterior legs |  |  |  |  |  |  |  |  |  |  |  |  |
| Lateral thigh-to-inguinal |  |  |  |  |  |  |  |  |  |  |  |  |
| Right | 1.88 | NV | NV | 0.17 | 0 | 0.29 | 0 | NV | ND | 0.55 | 0.44 | NV |
| Left | 0.59 | NV | ND | 0.50 | 0 | 0.48 | 0 | NV | ND | 0.82 | 0.44 | NV |
| Medial thigh-to-inguinal |  |  |  |  |  |  |  |  |  |  |  |  |
| Right |  |  |  | 0.33 | 0.38 | 0.48 | 0 | NV | ND | ND | ND | ND |
| Left |  |  |  | 0.33 | 0.25 | 1.25 | STR | 1.25 | ND | ND | ND | ND |
| Inferior |  |  |  |  |  |  |  |  |  |  |  |  |
| Right | 0.53 | 0.96 | 0.75 | 1.04 | 0.75 | 1.79 | 1.05 | 1.22 | 1.23 | 0.85 | 0.65 | 0.72 |
| Left | 0.72 | 0.72 | 0.69 | 0.45 | 0.87 | 0.89 | 1.62 | 0.77 | 0.89 | 0.85 | 0.39 | 0.72 |

ND = Not done; data not obtained, given the time constraints; NV = not visible; STR = streaming.
