# Supplementary material for: Case Report: The effect of automated manual lymphatic drainage therapy on lymphatic contractility in 4 distinct cases
Source: Front Med Technol. 2024 Jul 17;6:1397561. doi: 10.3389/fmedt.2024.1397561 (PMC11292613; doi:10.3389/fmedt.2024.1397561)
Supplement: Supplementary file 4 [file Table2.docx]

Table 2. Posterior pulsatile frequencies (pulses/minute) captured by near-infrared fluorescence lymphatic imaging before, during, and after treatment (txt) with automated manual lymphatic drainage therapy on the back of the body. Injection sites are listed in top down order as depicted in Figure 1. Multiple values per cell represent multiple vessels emanating from injection site. Empty cells indicate data were not applicable.

| **Anatomic Injection Site** | **Case 1, pulses/min** | | | **Case 2, pulses/min** | | | **Case 3, pulses/min** | | | **Case 4, pulses/min** | | |
| --- | --- | --- | --- | --- | --- | --- | --- | --- | --- | --- | --- | --- |
|  | Pre-txt | Inter-txt | Post-txt | Pre-txt | Inter-txt | Post-txt | Pre-txt | Inter-txt | Post-txt | Pre-txt | Inter-txt | Post-txt |
| Back |  |  |  |  |  |  |  |  |  |  |  |  |
| 1^st^ superior  back |  |  |  |  |  |  |  |  |  |  |  |  |
| Right | 1.20, 1.20 | ND | 1.06, 0.85 | 0.98 | ND | 0.50 | 0 | ND | 0 | 0.81 | ND | NV |
| Left | 1.55 | ND | 0.53 | 1.28 | ND | 0.92 | 0 | ND | 0 | 0.95 | ND | 1.80, 0.78 |
| 2^nd^ superior  back |  |  |  |  |  |  |  |  |  |  |  |  |
| Right |  |  |  |  |  |  | 0 | ND | 0 |  |  |  |
| Left |  |  |  |  |  |  | 0 | ND | 0 |  |  |  |
| Posterior thoracic  area |  |  |  |  |  |  |  |  |  |  |  |  |
| Right | 1.08 | ND | 0.85 | 0.39 | ND | 1.17 | 0.46 | ND | 0.60 |  |  |  |
| Left | 1.20 | ND | 0.95 | 0.69 | ND | 0.42 | 1.94 | ND | 1.07 |  |  |  |
| 1^st^ lumbar area |  |  |  |  |  |  |  |  |  |  |  |  |
| Right | STR | ND | 0.53 | 1.28 | ND | 0.42 | 1.08 | ND | 0.60 | 0.68 | ND | 1.48 |
| Left | 1.55, 0.84 | ND | 1.59, 0.84 | 1.08 | ND | 0.50 | 0 | ND | NV | 0.68 | ND | 0.87 |
| 2^nd^ lumbar area |  |  |  |  |  |  |  |  |  |  |  |  |
| Right |  |  |  |  |  |  | 0.54 | ND | 0.36 | 1.63 | ND | 1.39 |
| Left |  |  |  |  |  |  | 0 | ND | NV | 0.95 | ND | 0.61 |
| 3^rd^ lumbar area |  |  |  |  |  |  |  |  |  |  |  |  |
| Right |  |  |  |  |  |  |  |  |  | 0.68 | ND | 0.52 |
| Left |  |  |  |  |  |  |  |  |  | 0.81 | ND | 1.04 |
| 4^th^ lumbar area |  |  |  |  |  |  |  |  |  |  |  |  |
| Right |  |  |  |  |  |  |  |  |  | NV | ND | NV |
| Left |  |  |  |  |  |  |  |  |  | 1.08 | ND | 0.61 |
| Posterior legs |  |  |  |  |  |  |  |  |  |  |  |  |
| Right | ND | ND | 0.66 |  |  |  |  |  |  |  |  |  |
| Left | ND | ND | 0.44 |  |  |  |  |  |  |  |  |  |

ND = Not done; data not obtained, given the time constraints; NV = not visible; STR = streaming.
